# Mechanistic Insights into the Hot-Spot Formation and Pyrolysis of LLM-105 with Different Void Defects: A ReaxFF Molecular Dynamics Study

**DOI:** 10.3390/molecules30143016

**Published:** 2025-07-18

**Authors:** Mengyun Mei, Zijian Sun, Lixin Ye, Weihua Zhu

**Affiliations:** Institute for Computation in Molecular and Materials Science, School of Chemistry and Chemical Engineering, Nanjing University of Science and Technology, Nanjing 210094, China; 3115857405@njust.edu.cn (M.M.); zijian.sun@njust.edu.cn (Z.S.); lixin715@njust.edu.cn (L.Y.)

**Keywords:** LLM-105 crystal, hot-spot formation, void defects, decomposition mechanism, reactive molecular dynamic

## Abstract

To investigate the influences of void defects of different sizes, molecular dynamics combined with ReaxFF-lg reactive force field was used to study the hot-spot formation mechanism and thermal decomposition behavior of 2,6-diamino-3,5-dinitropyrazine-1-oxide (LLM-105) crystals with different void defects at 2500 K. The results indicate that larger void defects are more conducive to the formation of hot-spots. The consistency of the trends in time evolution of the potential energy, species numbers, and small molecules amounts between the ideal and void-containing LLM-105 crystals demonstrates that the presence of the void defect does not alter the decomposition mechanism of the LLM-105 molecule. An increase in the size of the void defect significantly increases the degree of diffusion of the C, H, O, and N atoms in the crystals, which affects the effective collisions between the atoms and thus alters the occurrence frequency of relevant reactions and the production of relevant products.

## 1. Introduction

Energetic materials, a class of metastable materials containing explosive groups, such as nitro, azide, hydrazide groups, etc. or oxidants and combustibles, have wide applications in military and civil fields [1,2,3,4]. They readily undergo rapid chemical reactions and release a large amount of energy when exposed to external stimuli, such as heat, impact, or friction [5,6,7]. Nevertheless, this could also lead to unexpected responses in energetic materials, which raises major safety concerns [8]. Therefore, the development of energetic materials with high energy and safety has become a topic of concern for scholars [9,10,11,12,13,14,15]. Many studies have shown that the factors that determine the safety performance of energetic materials include intrinsic structure, physical state, condensed form, crystal defects, etc. [16,17,18,19,20,21,22]. Among them, crystal defects, such as inclusions, dislocations, and grain boundaries, are often main factors affecting the sensitivity of energetic materials under external stimuli [23,24,25,26]. Therefore, it is necessary to explore the influence of internal defects on the structure and properties of energetic materials.

2,6-Diamino-3,5-dinitropyrazine-1-oxide (LLM-105), first synthesized by Katritzky et al. [27] in 1995, has remarkable properties, such as low sensitivity and high energy density, making it a very promising high energy density compound [28,29,30]. It is inevitable that some defects will be formed during the crystallization process of the LLM-105 crystal. Consequently, it is imperative to investigate the effects of defects on the physical and chemical properties of the LLM-105 crystal. Yu et al. [31] investigated the thermal decomposition process of LLM-105 and its gas products by using typical thermogravimetric (TG), differential scanning calorimetry (DSC), and Raman spectroscopy. Their findings indicate that low-dimensional defects, including twins, dislocations, and vacancies within the LLM-105 crystal, can trigger its initial decomposition. In order to investigate the relationship between internal voids and properties in ultrafine LLM-105 particles, Xing et al. [32] not only characterized the structure of internal nano-voids in the LLM-105 particles by using the CV-SAXS method but also probed its thermal stability by isothermal DSC experiments. Mei et al. [33] used density functional tight-binding molecular dynamics (DFTB-MD) to investigate the effects of the vacancy defects on the volume, electronic structure, energy, and initial decomposition mechanism of the LLM-105 crystal. It was found that the presence of the vacancies not only results in a reduction of the crystal size of LLM-105 but also accelerates the decomposition of the LLM-105 molecules. Unfortunately, the studies on the effects of the defects on the thermal decomposition of the LLM-105 crystal are relatively scarce compared to other common explosive crystals.

Most existing studies primarily involve two-dimensional defects, such as twins, dislocations, and vacancies. However, there is a notable lack of literature addressing the impact of three-dimensional void defects on the thermal decomposition mechanisms of explosives. Fortunately, theoretical simulations have been widely used to investigate the effects of defects on the electronic structure, mechanical properties, thermal decomposition mechanisms, and hot-spot formation of the explosives [34,35,36,37,38,39,40,41]. Among them, the ReaxFF molecular dynamics (RMD) technique has also been widely used to investigate the effects of three-dimensional defects on the thermal decomposition mechanisms of the explosives. Tan et al. [42] investigated the effects of the spatial distribution of void defects on hot-spot formation and the temperature of 1,3,5-trinitroperhydro-1,3,5-triazine (RDX) crystals under shock stimulation based on RMD combined with the multiscale shock technique (MSST). Compared with the RDX crystals with a more dispersed distribution of voids, the crystals with a more homogeneous distribution of voids are more likely to combine multiple hot-spots into a larger hot-spot, thus promoting chemical reactions more efficiently. Sun et al. [43] investigated the effects of the voids with varying sizes on the hot-spot formation and decomposition mechanisms of 4,40,5,50-tetranitro-2,20-bi-1H-imidazole/2,4,6-triamino-5-nitropyrimidine-1,3-dioxide (TNBI/TANPDO) cocrystal by RMD simulations. They found that the presence of the holes promotes the formation of hot-spots in defective regions and accelerates chemical reactions. The nanoscale defects can increase the temperature in the local area around the voids. Zhou et al. [44] performed ReaxFF MD simulations to reveal the collapse dynamics and hot-spot formation mechanisms of octahydro-1,3,5,7-tetranitro-1,3,5,7-tetrazocine (HMX) crystals with nanovoids under impact, which deepened the understanding of the mechanisms that the void defects influence explosive detonation.

**Figure 1:**
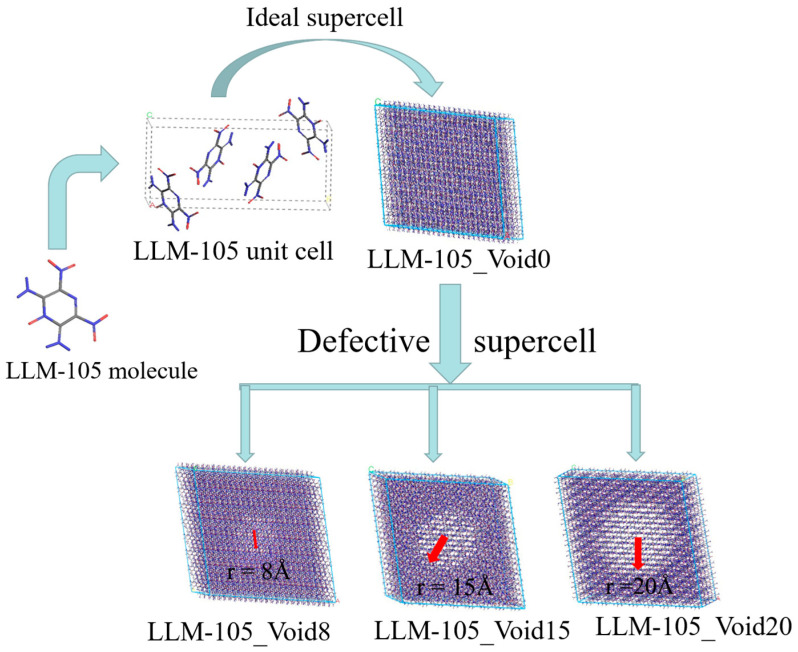
Unit cell, 12 × 4 × 8 ideal supercell model (LLM-105_Void0), and void-contained supercells (LLM-105_Void8, LLM-105_Void15, and LLM-105_Void20) of LLM-105 crystal.

In this work, we constructed a series of LLM-105 crystal models containing void defects with different sizes (Figure 1). Then, we investigated the effects of the voids of different sizes on the hot-spot formation and decomposition mechanisms of the LLM-105 crystal at high temperatures (2500 K) using the molecular dynamics method with the ReaxFF-Lg force field. The aim of our study is to reveal how the void defects affect the formation of hot-spots and decomposition mechanisms of explosives.

## 2. Results

### 2.1. Density Evolution

**Figure 2:**
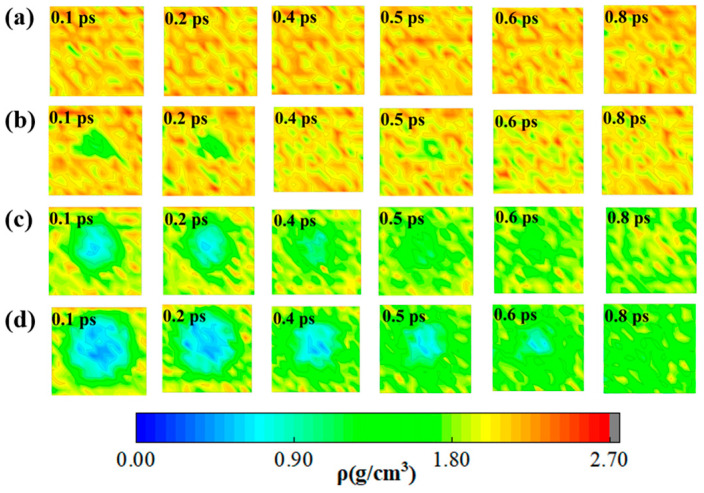
Early distributions of the densities of LLM-105_Void0 (**a**), LLM-105_Void8 (**b**), LLM-105_Void15 (**c**), and LLM-105_Void20 (**d**) at different times.

It is well known that the density distribution is usually one of the most important properties in structural characterization [45]. In order to clearly characterize the differences in atomic distribution and void evolution between defective and ideal systems during the decomposition process, we calculated the density profiles for the four systems along the XY-plane for the first 0.8 ps using the Visual Molecular Dynamics (VMD) [46] program in conjunction with a Density Calculator. The corresponding densities were obtained by slicing in the XY-plane, then calculating the mass in each slice, and dividing the density in each slice by the volume of the slice. Figure 2 illustrates the evolution of the densities of different systems over time during the thermal decomposition process.

It is evident that the color of the density profile remains relatively constant over time for the ideal system. However, the color of the density profile of the defect systems undergoes a gradual transition over time. During 0.1–0.4 ps, the color of the density profile of the defect systems exhibits a distinct gradient evolution, changing from blue at the center to green and finally to yellow in the peripheral regions. This indicates that, at the beginning of the reaction, the density of the defect-contained systems gradually decreases from the edge region to the center. The region with the lower density distribution in the center region represents the initial void region. As the reaction proceeds, the blue and green regions gradually shrink, while the yellow region expands. However, the edges of the yellow region become progressively lighter in color. In addition, we observed a gradual convergence of the lower density region at the center with time evolution. These phenomena suggest that the voids were gradually filled by their surrounding atoms. The surrounding atoms or molecules diffuse into the void region. This phenomenon can be explained using Fick’s law of diffusion [47,48]:

(1) $$J=-D\frac{d_{c}}{d_{x}}$$

where *J* is the one-dimensional flow in moles or grams per area per unit time across a reference plane, *D* is the diffusion coefficient, and *c* is the concentration in moles or grams per unit volume. The formula indicates that atoms within the system diffuse from high-density regions toward low-density regions. Due to the lower density of void regions, atoms from surrounding high-density zones migrate toward these voids. As a result, the density in the void areas gradually increases, and the surrounding atoms progressively converge into the void areas. In addition, the chemical potential (*μ*) is higher in the high-density region and lower in the low-density region. Diffusion is driven by the chemical potential gradient until *μ* becomes uniform (equilibrium state).

By 0.8 ps, the density distribution for the system becomes more even, suggesting that the atoms in the hole region have been saturated by its surrounding atoms.

### 2.2. Evolution of Hotspots and Maximum Temperature

**Figure 3:**
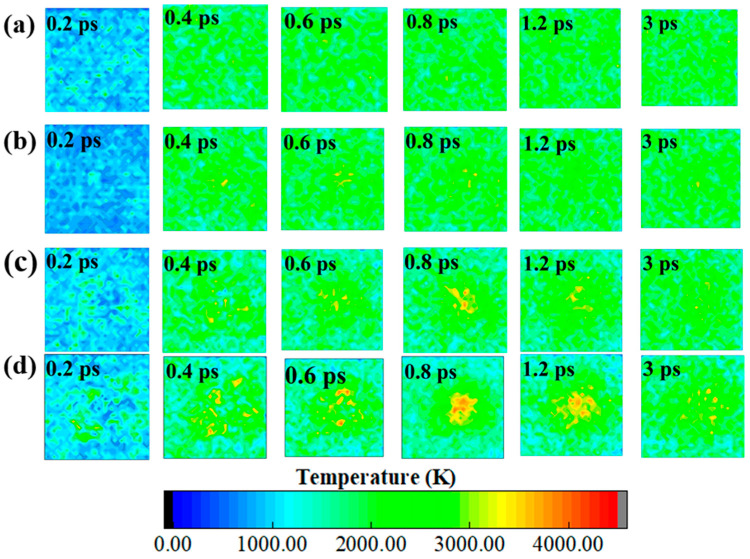
Early local distributions of the temperatures of LLM-105_Void0 (**a**), LLM-105_Void8 (**b**), LLM-105_Void15 (**c**), and LLM-105_Void20 (**d**) at different times.

To more precisely observe the high-temperature formation process and evolution mechanism of the defect region in the LLM-105 crystal, the simulation systems were subdivided into 45 equal volume boxes [44] (three-dimensional grids) according to the energy equipartition theorem. This ensures that each box is statistically representative. Subsequently, the average temperature of the atoms in each box was calculated, and the results were projected along the cross-section perpendicular to the Z-axis. Finally, the 3D temperatures were compressed into a 2D distribution. As demonstrated in Figure 3, the temperature profiles of both ideal and defective systems during the initial thermal decomposition at 2500 K were depicted. In addition, there is a transition from blue to green in the visualized region in the four systems. There are yellow areas in the LLM-105_Void8, LLM-105_Void15, and LLM-105_Void20. These yellow regions are predominantly located within the void regions. These phenomena indicate that the temperature of the system gradually increases and the temperatures of the void regions become higher during thermal decomposition compared to other regions, which are often referred to as “hot-spots” [43,49,50]. Johnson et al. [51] observed the formation of a hot-spot in an HMX crystal containing a void defect during impact compression. The temperature of this hot-spot was 4000 K, which proves the reliability of the hot-spot formation mechanism and temperature peaks in this work.

As seen in Figure 2, within a range of 0–0.6 ps, the atoms surrounding the voids in LLM-105_Void8, LLM-105_Void15, and LLM-105_Void20 gradually dispersed into the void area. Zhang et al. [52] proposed that the continuous influx of particles into the void induces successive collision events and chemical reactions, resulting in the release of heat. This leads to a gradual increase in the temperature of the void region, which is responsible for the formation of the hot-spot. In addition, we observed that the high-temperature region gradually increases as the size of the void increases, suggesting that an increase in the size of the void favors the formation of a hot-spot. In addition, it is observed that the green area gradually increases in size and gradually covers the original blue area. This indicates that the high temperature in the central region gradually spreads to the surrounding area. The larger the size of the void, the longer the time takes for the high temperature in the central region to spread to the surrounding area until the surrounding temperature gradually increases. This takes more time for the central atoms to collide. In addition, as the surrounding atoms gradually gather towards the void, the larger void will hold more particles, thus increasing the probability of collisions between the particles in the central region and generating more heat. These are reasons why the hot-spots also increase with the larger void sizes. As illustrated in Figure 3b,d, the distribution of yellow areas undergoes a transition from dispersion to aggregation from 0.4 to 0.8 ps; the yellow area at 0.8 ps was concentrated in the central area, while from 0.8 to 3 ps, the yellow area gradually dispersed until it disappeared. These findings suggest that the formation of the hot-spots in the void-contained systems initiates in the surrounding region of the voids during the thermal decomposition process. These hot-spots then gradually shrink to the central region before finally disappearing.

**Figure 4:**
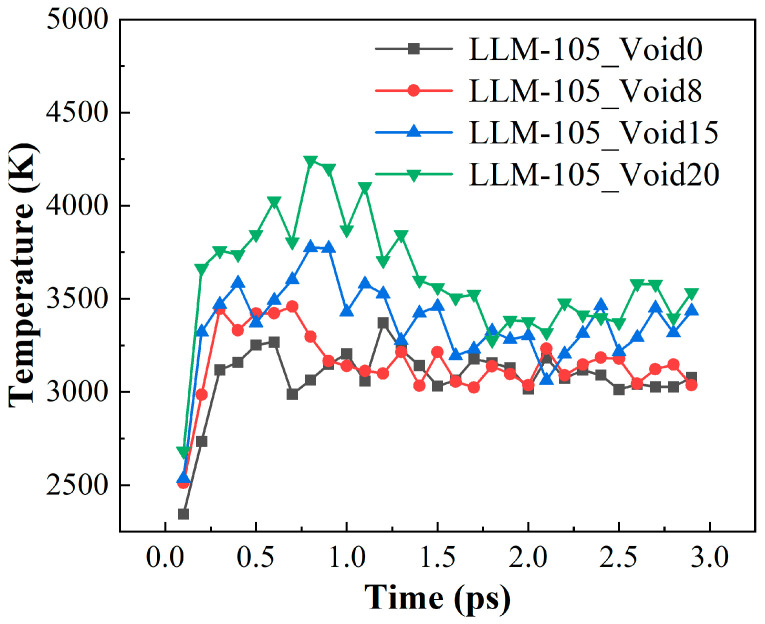
Evolution of the maximum temperature for the four systems over time.

To quantitatively measure the temperature differences in the hot-spot region for different systems, we plotted the time evolution of the maximum temperature for the four systems in 0–3.0 ps, as shown in Figure 4. From Figure 4, it can be seen that the temperature peaks first increase and then decrease for all the systems and finally stabilize after 2.0 ps. This is consistent with the formation process of the aforementioned hot-spots. The gradual increase of the maximum value of the system’s temperature can be considered to represent the formation process of the hot-spot area from scratch. Secondly, the process of gradually decreasing the maximum temperature corresponds to a gradual decrease in the hot-spot area until it completely disappears. The balance of the final maximum temperature suggests that the temperature of the system begins to be distributed evenly. The maximum temperatures and the time of their appearance for LLM-105_Void0, LLM-105_Void8, LLM-105_Void15, and LLM-105_Void20 are 3370.25 K (1.2 ps), 3459.07 K (0.7 ps), 3774.89 K (0.8 ps), and 4243.45 K (0.8 ps), respectively. These results demonstrate that the presence of the void defect increases the temperature peak of the system. It is evident that there is a direct correlation between the increase in the maximum temperature and the rise in the void ratio. Concurrently, the presence of the void defects can expedite the temperature of the system to reach a peak. Furthermore, the maximum temperatures of LLM-105_Void8, LLM-105_Void15, and LLM-105_Void20 occurred at around 0.8 ps. This is consistent with the time at which the yellow area (hot-spot) in Figure 3 begins to cluster in the central region. This indicates that the presence of the void defects can promote the formation of the hot-spots. The increase in the size of the void can lead to an increase in the maximum temperature.

### 2.3. Early Evolution of Kinetic Energy

**Figure 5:**
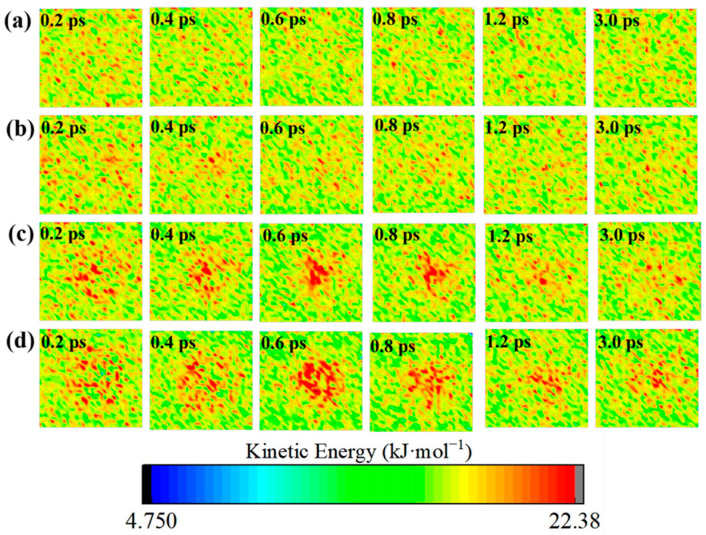
Early local distributions of the kinetic energy of LLM-105_Void0 (**a**), LLM-105_Void8 (**b**), LLM-105_Void15 (**c**), and LLM-105_Void20 (**d**) at different times.

To describe the formation mechanism of the hot-spot, we obtained the kinetic energy distribution cloud map in the same way as the temperature distribution cloud map described above. Figure 5 shows the kinetic energy distribution of the four systems. Clearly, the kinetic energy distribution of the ideal system is very homogeneous. For the defect systems, the kinetic energy of the void area is greater than that of the surrounding area. The evolution of the local kinetic energy is also consistent with the evolution of the local temperature. In the defective systems, the larger kinetic energy values are initially distributed near the void areas and then gradually converge towards the center. The maximum value of the kinetic energy in the central region occurred at 0.6–0.8 ps. The maximum temperature of the void systems also occurred at this time. These phenomena suggest that the kinetic energy transfers during the thermal decomposition of the void system are responsible for the formation and gradual evolution of the hot-spots over time.

### 2.4. Early Evolution of LLM-105 Molecules

**Figure 6:**
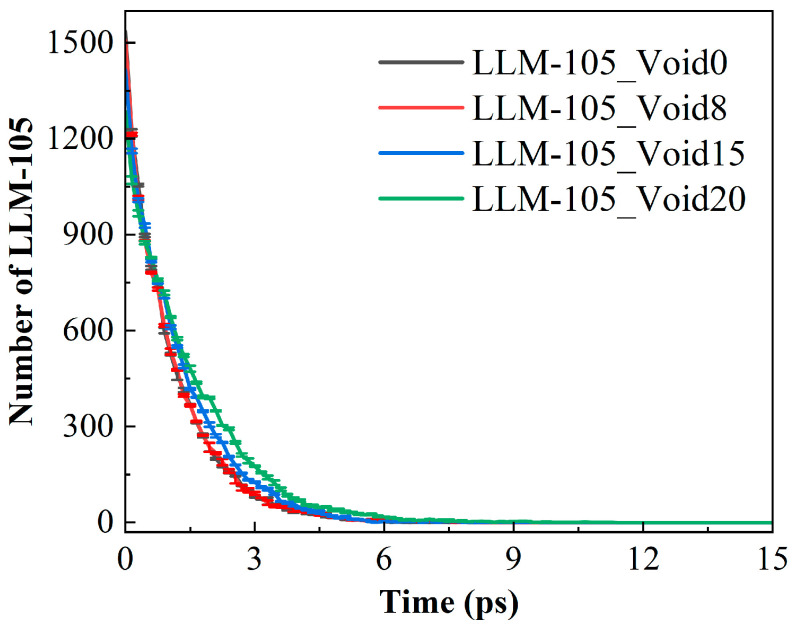
Evolution of the number of LLM-105 molecules in the four systems over time during the initial decomposition process.

**Figure 7:**
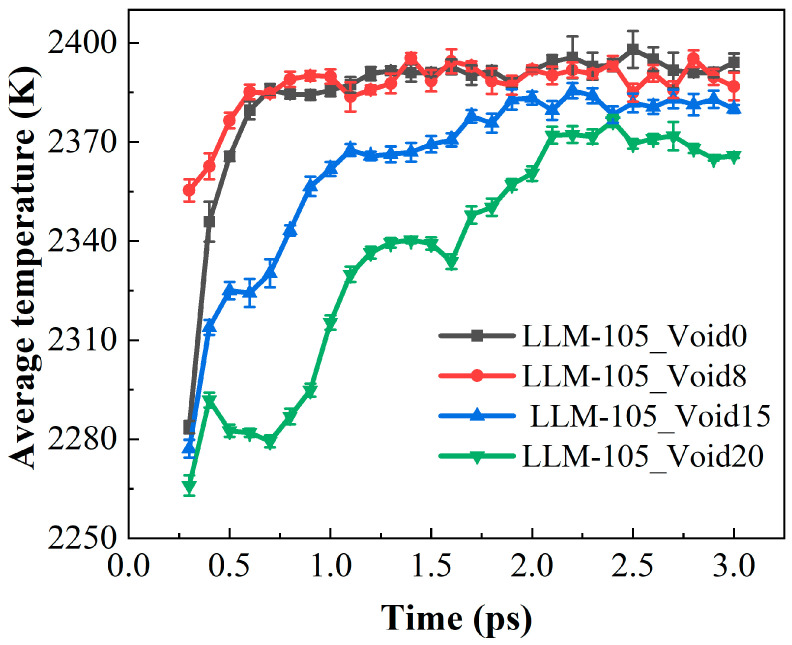
Evolution of the average temperature for the four systems over time.

Figure 6 illustrates the evolution of the number of LLM-105 molecules for these four systems during the initial reaction phase. In the four systems, the LLM-105 molecules completely decomposed within 15 ps. Notably, the decay rates of the LLM-105 molecules exhibited distinct variations among these systems, particularly within 0–3 ps. In order to describe the complete decomposition of the explosive molecules, we have statistically analyzed the time for the complete decomposition of the LLM-105 molecules in the four systems. The times for the complete decomposition of the LLM-105 molecules in the four systems are 7.8, 8.1, 9.45, and 11.4 ps, respectively. The presence of the voids delayed the complete decomposition time of the LLM-105 molecules. This indicates that the presence of the voids is not conducive to the decomposition of the LLM-105 molecule. This phenomenon has also been reported in relevant experiments by Xing et al. [32]. When the internal void size of LLM-105 increases to approximately 13 nm, its thermal decomposition time extends from 31 to 40 min. This phenomenon is essentially the result of the formation of the hot-spot. As shown in Figure 3, the region around the hot-spot in the void system has a significantly lower temperature than that in the ideal system. To quantitatively evaluate the temperature of the system, we calculated the average temperature of the four systems in 0–3 ps. The corresponding results were shown in Figure 7. As can be seen in Figure 7, the average temperature of the void systems is significantly lower than that of the ideal system. Temperature is often an important factor determining the thermal decomposition of explosive molecules. The key factor determining the delay in the complete decomposition of the LLM-105 molecule is the lower average temperature in the void systems.

### 2.5. Evolution of Potential Energy and Number of Species

**Figure 8:**
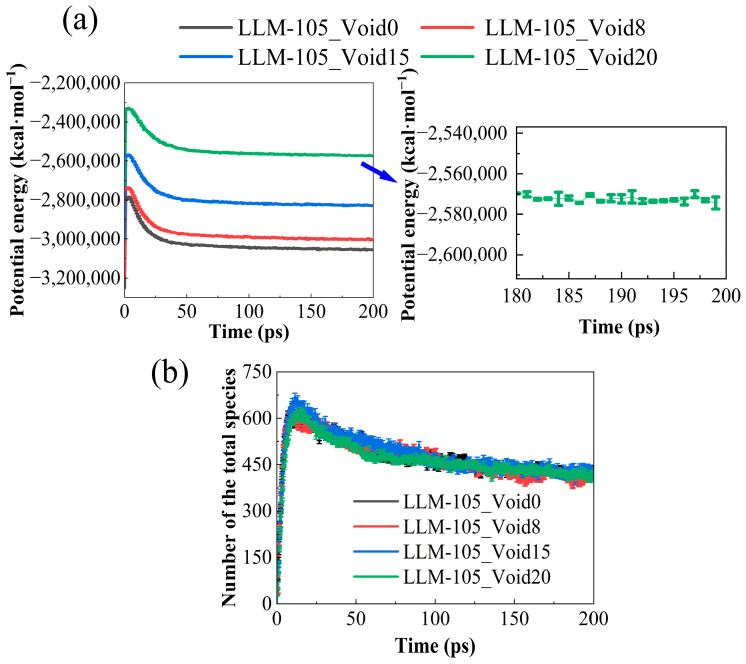
Time evolution of the potential energies (**a**) and the number of total species (**b**) for the four systems.

The evolution trend of the potential energy curve can reflect the reaction process and the equilibrium state of the system. Figure 8a shows the trend of the potential energy evolution with time for the ideal and defect-contained systems. As shown in Figure 8a, at the beginning of the reaction, the potential energy of the system shows a rapid increase as the time increases. Then, the potential energy reaches its maximum. After that, the potential energy of the system starts to decrease. This is due to the rapid decomposition of the LLM-105 molecule in the system. At that time, secondary reactions occur among initial decomposition products. As a result, a large number of intermediates and stable small molecule products will continue to be produced. The exothermic rate of the system will be much higher than the endothermic rate, and the potential energy will therefore continue to decrease. Eventually, the potential will gradually equalize, indicating that the chemical reaction is in an equilibrium. The trend in the evolution of the potential energy here is consistent with the thermal decomposition behaviors of LLM-105 as reported in previous studies [53,54]. Thus, the presence of the void defects does not change the thermal decomposition process of the systems. Interestingly, the potential energy values of these defective systems all reached their peaks around 3.0 ps., coinciding with the time that the hot-spot begins to disappear and the temperature of the system becomes uniform.

Figure 8b displays the time evolution of the number of species during the thermal decomposition of the four systems. As shown in Figure 8b, the evolution of the number of species over time increases and then decreases. In the later stages of the reaction, the number of species are very close for the four systems. Since the number of atoms in LLM-105_Void8, LLM-105_Void15, and LLM-105_Void20 at different times is significantly smaller than that in LLM-105_Void0, the final number of species is very close to that in LLM-105_Void0, suggesting that the presence of the void defects facilitates the decomposition of the systems.

### 2.6. Pyrolysis Mechanisms and Chemical Species

The main reactions and their frequencies for different systems within 200 ps were counted to investigate the effects of the voids on the thermal decomposition mechanisms of the LLM-105 crystal. Firstly, the frequencies of direct removal of NH_2_, NO_2_, H, NO_2_, and O were recorded. Table 1 displays five corresponding reaction paths and reaction frequencies: path A (C_4_H_4_O_5_N_6_ → C_4_H_2_O_5_N_5_ + NH_2_), path B (C_4_H_4_O_5_N_6_ → C_4_H_4_O_4_N_5_ + NO), path C (C_4_H_4_O_5_N_6_ → C_4_H_3_O_5_N_6_ + H), path D (C_4_H_4_O_5_N_6_ → C_4_H_4_O_3_N_5_ + NO_2_), and path E (C_4_H_4_O_5_N_6_ → C_4_H_4_O_4_N_6_ + O). Among them, the frequency of path D is highest. This indicates that the reaction of removing the nitro group is dominant in the thermal decomposition of the LLM-105 molecule. Furthermore, the frequencies of paths A~D can be ordered as A < B < C < D for both the ideal and defective systems. This is an indication that the presence of the void defect does not alter the decomposition mechanism of the LLM-105 molecule. However, the presence of the void defect significantly increases the frequency of these four reaction paths. The frequency of both paths B and D for all four systems can be ranked as LLM-105_Void20 > LM-105_Void15 > LLM-105_Void8 > LLM-105_Void0. This suggests that the larger the void size is, the higher the occurrence frequencies of paths B and D are. In path E, the presence of the void defect prevents the removal of the O atom from the LLM-105 molecule. The larger the void size is, the less likely this reaction will take place.

We have calculated the bond dissociation energy (BDE) of two paths, A and D, in the systems using density functional theory (DFT) (Table S3 of the Supporting Information). In all systems, the BDE value of path A is significantly greater than that of D, in agreement with our RMD-simulated results that the frequency of path A is lower than that of path D. In addition, for these two reactions, the BDE values of the void systems are lower than those of the ideal system, in accordance with our RMD-simulated results that the reaction frequencies of the void systems are lower than those of the ideal system. This demonstrates the reliability of our RMD simulations.

Table 2 lists the reaction pathways of some small molecular products obtained during the whole decomposition process. Their small molecules were produced in different decomposition paths, which have different occurrence frequencies in the four systems. However, the presence of the void defects can significantly accelerate the reaction pathways HNO → H + NO, HNO_2_ → OH + NO, H + N_2_H → N_2_ + H_2_, and OH + HNO → H_2_O + NO. These reactions occur more frequently in the systems with higher concentrations of the void defect. However, the presence of the void defect suppresses the occurrence of HN_2_O → OH + N_2_ and H + OH → H_2_O. In summary, the presence of the void defect alters the frequency of relevant reactions occurring in the systems. These effects can either facilitate or inhibit these decomposition paths. This is similar to the phenomenon observed by Zhou et al. [55] in studying the effects of the vacancy defects on the thermal decomposition mechanisms of HMX. Their work demonstrated that the presence of the vacancy defect accelerates the process of N-N bond cleavage and the ring-opening reaction but inhibits the formation of HONO.

**Figure 9:**
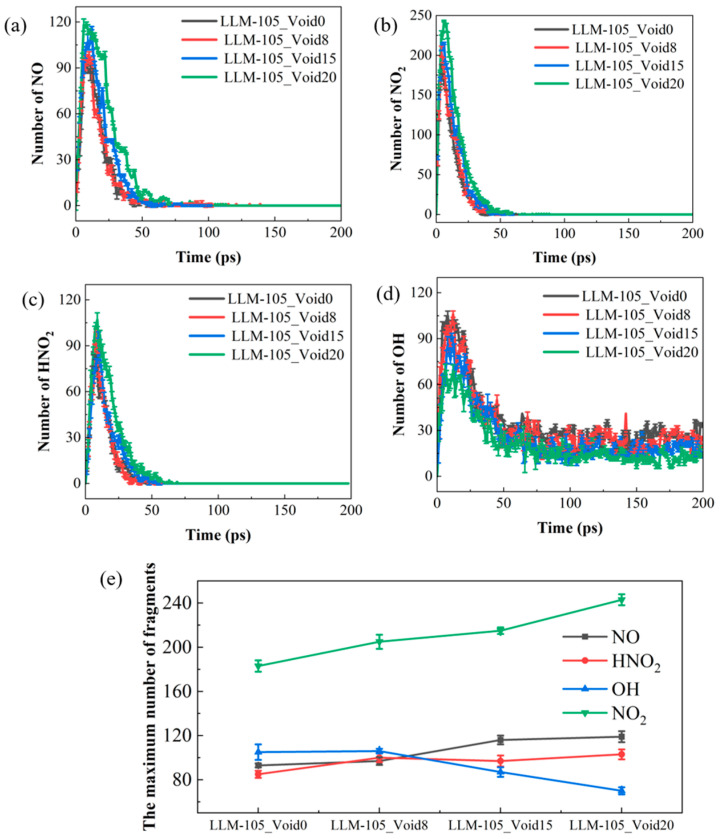
Time evolution of the number of NO, NO_2_, HONO, and OH molecules (**a**–**d**) and the maximum values of these yields (**e**) for the four systems.

Through quantitative analysis of the thermal decomposition products, it is found that CO_2_, H_2_O, N_2_, H_2_, HNO_2_, NO_2_, NO, and OH can be formed during the decomposition of the four systems at 200 ps. Statistically, the yields of HNO_2_, NO_2_, NO, and OH decrease as the reaction proceeds, so they are regarded as intermediaries. CO_2_, H_2_O, N_2_, and H_2_ are classified as final products due to their stability in the later stages of the reaction. Figure 9a–d shows the evolution of the number of intermediate products with time during the thermal decomposition of the four systems. The number of intermediate products increases at the beginning of the decomposition, reaches a peak, and then gradually decreases. The reason why these substances gradually decrease and disappear in the later stage is that HNO_2_ and NO_2_ decompose into NO and OH, and then, NO and OH participate in the formation of a large amount of H_2_O and N_2_. Among these products, the hydroxyl radical (OH) stands out as particularly unusual due to its persistently high concentration in the late stages of the decomposition. Yuan et al. [56] pointed out that this phenomenon is normal because the reaction H + OH → H_2_O is reversible at high temperatures. This reversible reaction leads to the recombination of hydrogen atom into H_2_, so there will be some free OH radicals in the systems. To distinguish the effects of the void defect on these products, the maximum number of various intermediates was plotted in Figure 9e. Notably, the presence of the void defect promotes the production of NO, NO_2_, and HNO_2_ but inhibits the production of OH, implying that the introduction of the void defect is favorable for the formation of the nitrogen-containing intermediate species. The maximum yields of NO, NO_2_, and HNO_2_ exhibit a positive dependence on the concentration of the void defect, while the maximum number of OH molecules presents an inverse relationship.

**Figure 10:**
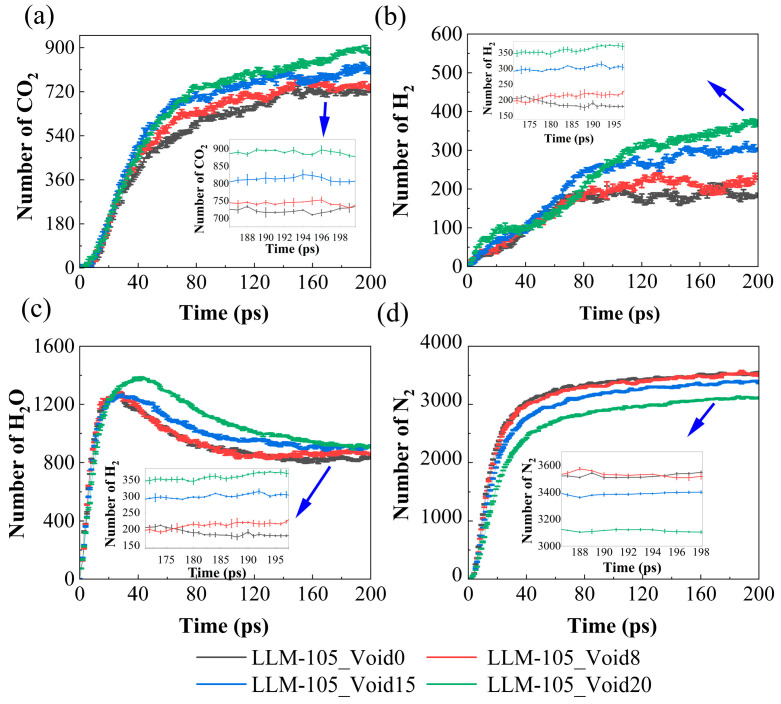
Time evolution of the number of final products of (**a**) CO_2_, (**b**) H_2_, (**c**) H_2_O, and (**d**) N_2_ in the four systems.

The evolution of the number of final products over time is shown in Figure 10. As seen in Figure 10a,b, the quantities of CO_2_ and H_2_ increase gradually with time in the four systems. Within the first 50 ps, the quantities of CO_2_ and H_2_ are essentially the same in the four systems. However, the presence of the void defect significantly accelerates the formation of these two products after 50 ps. The amounts of CO_2_ and H_2_ for the four systems increase in the following order: LLM-108_Void20 > LLM-105_Void15 > LLM-105_Void8 > LLM-105_Void0. In Figure 10c, the yield of H_2_O in the four systems first increases, then decreases, and finally tends to equilibrate. Within 40–120 ps, the yield of H_2_O follows a trend of LLM-105_Void20 > LLM-105_Void15 > LLM-105_Void8 ≈ LLM-105_Void0. After 160 ps, the sizes of the void defects have less effect on the yield of H_2_O. This indicates that the enhancement of the number of H_2_O molecules affected by the void defects is mainly concentrated in the range 40–120 ps. The H_2_O molecules will participate in secondary chemical reactions as reactants. This leads to a slight decrease in its number until a final equilibrium. The amount of N_2_ increases and then remains in equilibrium, as shown in Figure 10d. During the initial 40 ps, there was almost no difference in the amount of nitrogen produced in the four systems. However, after 40 ps, the presence of the void defects significantly suppressed the production of N_2_. The results here are in agreement with the findings by Sun et al. [43]. It has been suggested that the presence of the void defects suppresses the production of N_2_. The number of N_2_ molecules in the four systems follows the trend of LLM-105_Void0 > LLM-105_Void8 > LLM-105_Void15 > LLM-105_Void20.

The main intermediates during the thermal decomposition of the LLM-105 molecules are NO_2_, NO, HONO, and OH, while the final products are CO_2_, H_2_O, N_2_, and H_2_. These results are consistent with the conclusions reported in the related literature [53,54], and the evolutionary trends of these products also remain consistent with those reported in the literature. Moreover, the removal of NO_2_ is the dominant reaction from the perspective of the reaction frequencies, which is also consistent with the dominant breakdown channel reported by Cheng et al. [57] during the decomposition of the LLM-105 molecules.

Our study indicates that these voids will lower the average temperature, thereby slowing down the decomposition process. However, they will also increase the occurrence frequency of certain reaction paths, facilitating the formation of final products, such as carbon dioxide and hydrogen. This may seem contradictory, but it is actually a reasonable phenomenon. Firstly, we have captured snapshots at 1 ps for these four systems (Figure S2 of the Supporting Information). Only small molecular products produced during the initial decomposition process were presented in Figure S2, such as NO_2_, NO, and OH. The yellow region represents small molecule products located within a 25 Å radius sphere centered at coordinates (33.608, 31.688, 27.046). Table S2 of the Supporting Information presents the total atomic count of all small molecule products (N_total_), the number of atoms localized within the yellow-highlighted region (N_25Å_), and the proportion of yellow-region atoms relative to the total small molecule population (N_25Å_/N_total_). It is worth noting that the total number of atoms of small molecular products in the system decreases with the increasing void size. This decrease is related to the decrease in the reaction rate constant of the system, which is mainly driven by the decrease in the mean temperature. Meanwhile, the ratio of the number of atoms in the yellow area of the void systems to the total number of atoms is significantly higher than this ratio in the ideal system. This trend suggests that the formation of the hot-spots significantly enhances the occurrence frequency of chemical reactions in the local region. Consequently, the presence of a hot-spot enhances the reaction frequency of associated chemical processes within its immediate vicinity (a localized domain of the overall system). In Table 1 and Table 2, although the presence of the voids changes the occurrence frequency of related reactions, the reactions C_4_H_4_O_5_N_6_ → C_4_H_4_O_3_N_5_ + NO_2_ and OH + HNO → H_2_O + NO are still dominant reactions in all of the systems, and the occurrence frequency of C_4_H_4_O_5_N_6_ → C_4_H_4_O_4_N_5_ + NO is also the lowest. In other words, the presence of the voids does not change the dominant decomposition pathway but only alters the number of relevant reaction pathways in the local region. In the first 3 ps, the average temperatures of the void systems are lower than those of the ideal system, but the former gradually approaches the latter. This indicates that, in the later stages of the reaction, the temperatures of the four systems will converge. When the mean temperature of the system is close, the factor that affects the chemical reactions in the system is not the temperature. In the later stages of the reaction, the presence of the voids increases the yields of CO_2_ and H_2_. This is because the free-moving spaces of the void systems are larger and the diffusion coefficients of the atoms in the void systems increase, conducive to effective atom collisions. The formation of chemical products is caused by efficient collisions of molecules and atoms. The probability of collisions between atoms and molecules in the void systems increases, as does the yields of CO_2_ and H_2_.

### 2.7. Diffusion Behavior

As the simulation time increases, the free radicals and small molecules produced during the decomposition will undergo further reactions. The prerequisite for these reactions to occur is effective collisions between the atoms. Therefore, it is particularly important to investigate the diffusion behaviors of different atoms in the systems. We fitted the root mean square displacements (MSDs) for these four systems and obtained their diffusion coefficients according to Einstein′s diffusion equation, Equation (2):

(2) $$D=\frac{1}{6}\underset{t\to \infty }{\lim}\frac{d}{dt}\langle {\left|\vec{r_{n}}\left(t\right)-\vec{r_{n}}\left(0\right)\right|}^{2}\rangle \;$$

where $\vec{r_{n}}\left(t\right)$ is the position of atom *n* at time *t*, and *D* is the diffusion coefficient (m^2^/s). 

**Figure 11:**
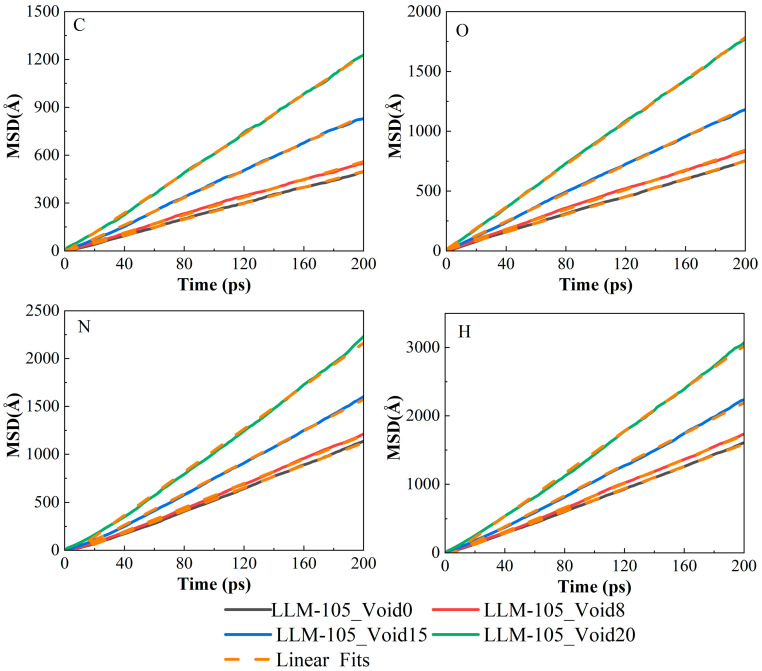
MSD curves of the C, O, N, and H atoms in the four systems over time.

Figure 11 shows the MSD curves of C, H, O, and N atoms of the LLM-105 molecules during the thermal decomposition of the four systems. The MSD curve for each atom shows an increasing trend with time. As the size of the void defect increases, the diffusion of the atoms becomes more pronounced. So, the presence of the void defect facilitates atomic diffusion. As the probability of atomic collisions increases, the occurrence likelihood of relevant reactions increases, thereby enhancing the numbers of corresponding molecules. The reason for the smaller MSD value during the initial reaction stage of individual atoms is due to the incomplete decomposition of the LLM-105 molecules. This will hinder the diffusion of the atoms. However, in the later stages of the decomposition, the LLM-105 molecules decompose completely, and the atoms and small radicals are easier to diffuse. The diffusion coefficients and the slopes of the MSD curves for these four atoms are listed in Table 3. The diffusion coefficients of the H atoms are the largest, followed by the N and O atoms, and those of the C atoms are the smallest, indicating that the H atoms are the most active during the thermal decomposition. The relative ordering of the diffusion coefficients for these four atoms remains consistent across all systems, following the sequence: LLM-105_Void20 > LLM-105_Void15 > LLM-105_Void8 > LLM-105_Void0. According to Formula (2), in three-dimensional space, the diffusion coefficient of atoms is directly proportional to the MSD. The slope of the MSD curve increases with the increase in the void size, which is consistent with the conclusion of the diffusion coefficients. Since the void models were obtained by subtracting multiple molecules from an ideal system, these removed molecules can provide the system with more space. As a result, the voids can provide additional free space and make atoms or molecules move easily, thus promoting the diffusion of atoms.

## 3. Computational Details

All molecular dynamics simulations were performed using the Large-scale Atomic/Molecular Massively Parallel Simulator (LAMMPS) [58] based on the ReaxFF-lg reactive force field [59]. The molecular dynamics simulations were performed using LAMMPS (version 2021), developed by Sandia National Laboratories under contract to the U.S. Department of Energy. The LAMMPS is an open-source software distributed under the GNU General Public License (GPL). As it does not have a commercial manufacturer, users must download and compile the code themselves or obtain it through a third-party platform. ReaxFF is a bond-order-dependent force field, and its connectivity is determined by the bond orders calculated from the interatomic distances that are updated at each iteration step. Thus, the ReaxFF force field can describe bond formation and dissociation during the simulation. The ReaxFF-lg force field energy expression is as follows:

(3) $$E_{Reax/lg}=E_{Reax}+E_{lg}\;$$

where *E_Reax_* represents the energy derived from the conventional ReaxFF force field [60]:

(4) $$\begin{array}{c} E_{Reax}\;=E_{bond}\;+\;E_{over}\;+\;E_{under}\;+\;E_{val}\;+\;E_{pen}\;+\\ E_{tors}\;+\;E_{conj}\;+\;E_{vdWalls}\;+\;E_{Coulomb}\; \end{array}$$

The energy terms in the formula are sequentially the bond energy term (*E*_bond_), the over-coordination energy correction term (*E*_over_), the under-coordination energy term (*E*_under_), the valence angle energy term (*E*_val_), the energy penalty for handling atoms with two double bonds term (*E*_pen_), the torsion angle energy term (*E*_tors_), the conjugated bond energies (*E*_conj_) term, the van der Waals energy term (*E*_vdW_), and the electrostatic energy (*E*_Comb_) term. And *E*_lg_ is the long-range-correction term using the low-gradient model. These terms together give the total energy (*E*_Reax-lg_).

Based on the crystal structure of LLM-105 with *a* = 5.709 Å, *b* = 15.84 Å, and *c* = 8.416 Å, *α* = 90.000°, *β* = 101.140°, *γ *= 90.000°, from the Cambridge Crystallographic Data Centre (CCDC) with CCDC numbers of 938,305, we constructed a 12 × 4 × 8 supercell of LLM-105. This ideal supercell was designated as LLM-105_Void0. Subsequently, by removing the atomic spheres with radii of 8, 15, and 20 Å centered at coordinates (33.608, 31.688, 27.046) from the LLM-105_Void0, we obtained three distinct void defect models with varying sizes, designated as LLM-105_Void8, LLM-105_Void15, and LLM-105_Void20, respectively. Figure 1 presents the structural schematics of these models along with the process for constructing the relevant models. The number of atoms in these four models is 29,814, 28,652, 26,904, and 24,434, respectively. The void ratios for these four models are 0%, 1.82%, 7.81%, and 16.28%, respectively.

The initial velocities of all atoms were set according to the randomly distributed Maxwell–Boltzmann law. We performed 20 ps MD simulations with the NPT ensemble at 298 K and 1 atm to equilibrate the four systems. Temperature and pressure were regulated using the Nosé-Hoover thermostat and Andersen barostat, respectively. Then we used MD to simulate their thermal decomposition processes with the NVT ensemble at 2500 K. The total duration was set to 200 ps with a time step of 0.1 fs. In the NPT simulations, the temperature damping parameter was set to 10 fs and the pressure damping parameter to 100 fs. The temperature damping parameter is set to 10 fs in the NVT simulations. Figure S1 of the Supporting Information shows the variation of the potential energy in 20 ps. After 20 ps of the NPT simulations, it is clear that the potential of the system approaches an equilibrium.

Table S1 of the Supporting Information presents the simulated lattice parameters of the LLM-105 crystal. These data are close to the experimental results, indicating that the ReaxFF-Lg force field is suitable for simulating the LLM-105 crystal. In addition, Jiang [54] and Lan [53] have also verified the applicability of ReaxFF-Lg force field to the LLM-105 crystal. Table S4 of the Supporting Information shows the parameter set of the ReaxFF-lg force field. ReaxFF-lg force field is a London dispersion term proposed in 2013 by Liu et al. [59], which also belongs to the long-range force correction term. The equation of state of the crystal structure obtained by fitting the modified reaction force field is in better agreement with the experimental values. As a result, the ReaxFF-Lg reaction force field has wide applications in the study of combustion and explosion of energetic materials, such as CL-20, TATB, HMX, and LLM-105. The ReaxFF-Lg reaction force field molecular dynamics is a highly powerful method to simulate complex chemical reactions, including exothermic processes. It handles exothermic reactions by dynamically adjusting bond orders and atomic interactions based on quantum mechanical principles, allowing bond breaking or formation and energy redistribution during the reactions [61]. A large number of literature reports have also proposed that the ReaxFF reaction force is suitable for handling the thermal decomposition process of such systems containing C, H, O, and N atoms [62,63,64]. The reaction analysis tool ReacNetGenerator [65] was utilized to characterize and track molecular species evolution throughout the simulations.

## 4. Conclusions

In this work, the influences of the void defects with varying porosities on hot-spot formation, decomposition mechanisms, and products of LLM-105 at 2500 K were investigated using the ReaxFF-lg molecular dynamics method. The results indicate that the atoms near the voids continuously diffuse into the void regions during the thermal decomposition process. The particle collisions and chemical reactions release a large amount of heat, leading to a gradual increase in the temperature within the voids and subsequent hot-spot formation. The process of hot-spot formation is generally as follows: The more dispersed high-temperature regions gradually converge towards the center, then form a hot-spot with the largest area, and finally the hot-spot gradually disappears as the distribution of atoms in the system becomes more homogeneous. Furthermore, an increase in the concentration of the void defect results in an increase in the maximum temperature in the hot-spot region, as well as an expansion of the area of the hot-spot region. Meanwhile, the presence of a void can also reduce the reaction rate constant of the LLM-105 molecules. The presence of void defects does not affect the decomposition mechanism of the LLM-105 molecule; however, it does influence the reaction frequency. The presence of void defects can promote the production of NO, NO_2_, HNO_2_, H_2_O, and CO_2_ and concurrently suppress the formation of OH and N_2_. These effects become increasingly pronounced with the rising concentrations of void defects.

## Figures and Tables

**Table 1:** Frequency of reactions A (C_4_H_4_O_5_N_6_ → C_4_H_2_O_5_N_5_ + NH_2_), B (C_4_H_4_O_5_N_6_ → C_4_H_4_O_4_N_5_ + NO), C (C_4_H_4_O_5_N_6_ → C_4_H_3_O_5_N_6_ + H), D (C_4_H_4_O_5_N_6_ → C_4_H_4_O_3_N_5_ + NO_2_), and E (C_4_H_4_O_5_N_6_ → C_4_H_4_O_4_N_6_ + O) during the four systems.

Primary Reactions		Occurrence Frequencies			
		LLM-105_Void0	LLM-105_Void8	LLM-105_Void15	LLM-105_Void20
A	C_4_H_4_O_5_N_6_ → C_4_H_2_O_5_N_5_ + NH_2_	19	21	33	27
B	C_4_H_4_O_5_N_6_ → C_4_H_4_O_4_N_5_ + NO	24	36	44	48
C	C_4_H_4_O_5_N_6_ → C_4_H_3_O_5_N_6_ + H	63	76	71	110
D	C_4_H_4_O_5_N_6_ → C_4_H_4_O_3_N_5_ + NO_2_	134	143	179	203
E	C_4_H_4_O_5_N_6_ → C_4_H_4_O_4_N_6_ + O	94	86	86	75

**Table 2:** Occurrence frequencies of some small molecular products during the decomposition process of the four systems.

Primary Reactions	Occurrence Frequencies			
	LLM-105_Void0	LLM-105_Void8	LLM-105_Void15	LLM-105_Void20
HNO → H + NO	109	133	142	229
HNO_2_ → OH + NO	65	67	88	160
H + N_2_H → N_2_ + H_2_	397	426	463	565
OH + HNO → H_2_O + NO	307	327	381	410
HN_2_O → OH + N_2_	244	246	206	148
H + OH → H_2_O	88	79	72	48
HNO_3_ → OH + NO_2_	55	63	57	69

**Table 3:** The slope of the MSD curve and diffusion coefficients (*D*/10^−8^ m^2^ s^−1^) of the C, O, N, and H atoms in the four systems.

	LLM-105_Void0	LLM-105_Void8	LLM-105_Void15	LLM-105_Void20
slope				
C	2.52	2.76	4.26	6.78
O	3.72	4.14	5.94	8.88
N	5.88	6.24	8.16	11.22
H	8.16	8.82	11.28	15.54
Diffusion coefficients				
C	0.42	0.46	0.71	1.13
O	0.62	0.69	0.99	1.48
N	0.98	1.04	1.36	1.87
H	1.36	1.47	1.88	2.59

## Data Availability

Data are contained within the article and Supplementary Materials.

## Supplementary Materials

The following supporting information can be downloaded at: https://www.mdpi.com/article/10.3390/molecules30143016/s1. Figure S1. The Potential energy with NPT simulation for 20 ps. Figure S2. Decomposition snapshots of different systems at 1 ps. Table S1. The lattice parameters of LLM-105 crystal. Table S2. The total number of atoms in the small molecule products at 1 ps (Ntotal), the number of atoms within a 25 Å radial cutoff from the central atom(N25Å) at 1 ps, and the proportion of the number of atoms within a 25 Å radial cutoff from the central atom to total number of atoms in the small molecule products (N25Å/Ntotal) at 1 ps. Table S3. The bond dissociation energy (BDE in kcal/mol) of path A and D. Table S4. The Reax-FF parameters for RMD simulation.
