# Supplementary material for: Mechanistic Insights into the Hot-Spot Formation and Pyrolysis of LLM-105 with Different Void Defects: A ReaxFF Molecular Dynamics Study
Source: Molecules. 2025 Jul 18;30(14):3016. doi: 10.3390/molecules30143016 (PMC12299919; doi:10.3390/molecules30143016)
Supplement: Supplementary file 1 [file molecules-30-03016-s001.zip › molecules-3731716-supplementary.pdf]

Supporting Information

for

Mechanistic Insights into the Hot-Spot Formation and Pyrolysis of LLM-105 with Different Void Defects: A ReaxFF MolecularDynamics Study

Mengyun Mei, Zijian Sun, Lixin Ye and Weihua Zhu *

Institute for Computation in Molecular and Materials Science, School of Chemistry and Chemical Engineering, Nanjing University of Science and Technology, Nanjing 210094, China
* Correspondence: zhuwh@njust.edu.cn

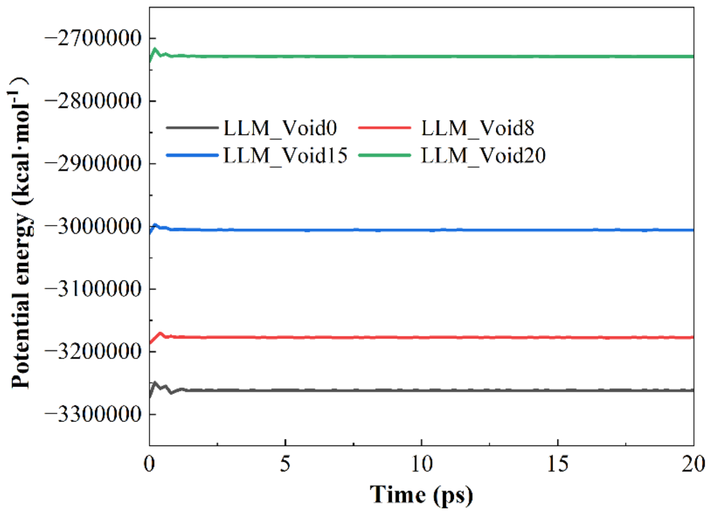

Figure S1. The Potential energy with NPT simulation for 20 ps.

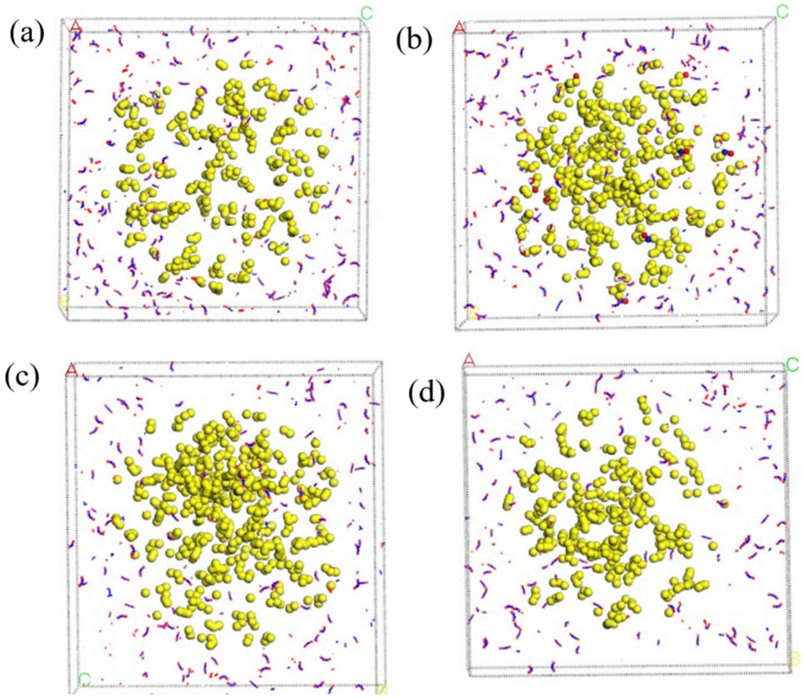

Figure S2. Decomposition snapshots of different systems at 1 ps.

Table S1. The lattice parameters of LLM-105 crystal.

	a (Å)	a (Å)	c (Å)	$\boldsymbol{\rho}$/(g.cm^{-3})	α	β	γ
Reference	68.508	63.376	67.328	1.922	90.000	101.140	90.000
ReaxFF/lg	67.785	62.708	65.363	2.033	90.000	101.140	90.000
Error (%)	3.3	1.0	2.9	5.7	0	0	0

Table S2. The total number of atoms in the small molecule products at 1 ps (N_{total}), the number of atoms within a 25 Å radial cutoff from the central atom($N_{25Å}$) at 1 ps, and the proportion of the number of atoms within a 25 Å radial cutoff from the central atom to total number of atoms in the small molecule products ($N_{25Å}$ /N_{total}) at 1 ps.

Model	$N_{25Å}$	N_{total}	$N_{25Å}$ /N_{total}
LLM-105_Void0	355	1347	26.35%
LLM-105_Void8	463	1307	35.42%
LLM-105_Void15	547	1283	42.63%
LLM-105_Void20	377	990	38.08%

Table S3. The bond dissociation energy (BDE in kcal/mol) of path A and D

Primary reactions	BDE			
	LLM-105_Void0	LLM-105_Void8	LLM-105_Void15	LLM-105_Void20
A	90.18	89.59	88.54	88.18
D	67.50	64.43	65.28	62.08

Table S4 The Reax-FF parameters for RMD simulation

```
Reactive MD-force field: nitramines (RDX/HMX/TATB/PETN))+innervdWaa+lg
 39       ! Number of general parameters
   50.0000 !Overcoordination parameter
    9.4514 !Overcoordination parameter
   29.8953 !Valency angle conjugation parameter
  216.5421 !Triple bond stabilisation parameter
   12.2245 !Triple bond stabilisation parameter
    0.0000 !C2-correction
    1.0701 !Undercoordination parameter
    7.5000 !Triple bond stabilisation parameter
   11.9083 !Undercoordination parameter
   13.3822 !Undercoordination parameter
  -10.9834 !Triple bond stabilization energy
    0.0000 !Lower Taper-radius
   10.0000 !Upper Taper-radius
    2.8793 !Not used
   33.8667 !Valency undercoordination
    3.3976 !Valency angle/lone pair parameter
    1.0563 !Valency angle
    2.0384 !Valency angle parameter
    6.1431 !Not used
    6.9290 !Double bond/angle parameter
    0.0283 !Double bond/angle parameter: overcoord
    0.0570 !Double bond/angle parameter: overcoord
   -2.4837 !Not used
    5.8374 !Torsion/BO parameter
   10.0000 !Torsion overcoordination
    1.8820 !Torsion overcoordination
   -1.2327 !Conjugation 0 (not used)
    2.1861 !Conjugation
    1.5591 !vdWaals shielding
    0.0100 !Cutoff for bond order (*100)
    4.8414 !Valency angle conjugation parameter
    3.5857 !Overcoordination parameter
   38.6472 !Overcoordination parameter
    2.1533 !Valency/lone pair parameter
    0.5000 !Not used
    1.0000 !Scale factor (d) in dispersion
    5.0000 !Molecular energy (not used)
    0.0000 !Molecular energy (not used)
    6.9784 !Valency angle conjugation parameter
  7    ! Nr of atoms; cov.r; valency;a.m;Rvdw;Evdw;gammaEEM;cov.r2;#
             alfa;gammavdW;valency;Eunder;Eover;chiEEM;etaEEM;n.u.
```

```
        cov r3;Elp;Heat inc.;n.u.;n.u.;n.u.;n.u.
        ov/un;val1;n.u.;val3,vval4
C    1.3742    4.0000   12.0000    1.9684    0.1723    0.8712    1.2385
     4.0000    8.7696  100.0000    4.0000   31.0823   79.5548    5.7254
     6.9235    0.0000    1.2104    0.0000  183.8108    5.7419   33.3951
     11.9957   0.8563    0.0000   -2.8983    4.7820    1.0564    4.0000
     2.9663    1.6737    0.1421   14.0707    0.0001    1.9255
H    0.6867    1.0000    1.0080    1.3525    0.0616    0.8910   -0.1000    1.0000
     9.1506  100.0000    1.0000    0.0000  121.1250    3.8446   10.0839    1.0000
     -0.1000   0.0000   58.4369    3.8461    3.2540    1.0000    1.0698
     0.0000  -15.7683    2.1504    1.0338    1.0000    2.8793    1.2669
     0.0139   12.4538    0.0001    1.4430
O    1.3142    2.0000   15.9990    1.9741    0.0880    0.8712    1.1139
     6.0000    9.9926  100.0000    4.0000   29.5271  116.0768    8.5000    7.1412
     2.0000    0.9909   14.7235   69.2921    9.1371    1.6258    0.1863
     0.9745    0.0000   -3.5965    2.5000    1.0493    4.0000    2.9225
     1.7221    0.1670   13.999   624.0000    1.7500
N    1.2456    3.0000   14.0000    2.0437    0.1035    0.8712    1.1911
     5.0000    9.8823  100.0000    4.0000   32.4758  100.0000    6.8453
     6.8349    2.0000    1.0636    0.0276  127.9672    2.2169    2.8632
     2.4419    0.9745    0.0000   -4.0959    2.0047    1.0183    4.0000
     2.8793    1.5967    0.1649   13.9888  1239.000    1.8300
S    1.9647    2.0000   32.0600    2.0783    0.2176    1.0336    1.5386    6.000
     9.9676    5.0812    4.0000   35.1648  112.1416    6.5000    8.2545
     2.0000    1.4703    9.4922   70.0338    8.5146   28.0801    8.5010
     0.9745    0.0000  -10.0773    2.7466    1.0338    6.2998    2.8793
     1.8000    0.0000   14.0000  180.0000    2.0783
Si   2.0276    4.0000   28.0600    2.2042    0.1322    0.8218    1.5758
     4.0000   11.9413    2.0618    4.0000   11.8211  136.4845    1.8038
     7.3852    0.0000   -1.0000    0.0000  126.5331    6.4918    8.5961
     0.2368    0.8563    0.0000   -3.8112    3.1873    1.0338    4.0000
     2.5791    0.0000    0.0000    0.0000  180.0000    2.2042
X   -0.1000    2.0000    1.0080    2.0000    0.0000    1.0000   -0.1000
     6.0000   10.0000    2.5000    4.0000    0.0000    0.0000    8.5000
     1.5000    0.0000   -0.1000    0.0000   -2.3700    8.7410   13.3640
     0.6690    0.9745    0.0000  -11.0000    2.7466    1.0338    4.0000
     2.8793    0.0000    0.0000    0.0000  180.0000    2.0000
18        ! Nr of bonds; Edis1;LPpen;n.u.;pbe1;pbo5;13corr;pbo6
                   pbe2;pbo3;pbo4;Etrip;pbo1;pbo2;ovcorr
1    1  141.9346  113.4487   67.6027    0.1554   -0.3045    1.0000   30.4515
          0.4283    0.0801   -0.2113    8.5395    1.0000   -0.0933    6.6967
          1.0000    0.0000
1    2  163.6889    0.0000    0.0000   -0.4525    0.0000    1.0000    6.0000
          0.5921   12.1053    1.0000    0.0000    1.0000   -0.0097    8.6351
```

		0.0000	0.0000					
2	2	169.8421	0.0000	0.0000	-0.3591	0.0000	1.0000	6.0000
		0.7503	9.3119	1.0000	0.0000	1.0000	-0.0169	5.9406
		0.0000	0.0000					
1	3	159.7219	116.8921	77.9315	-0.4324	-0.1742	1.0000	15.0019
		0.5160	1.2934	-0.3079	7.0252	1.0000	-0.1543	4.5116
		0.0000	0.0000					
3	3	108.9631	158.3501	42.0558	0.1226	-0.1324	1.0000	28.5716
		0.2545	1.0000	-0.2656	8.6489	1.0000	-0.1000	6.8482
		1.0000	0.0000					
1	4	128.9104	171.2945	100.5836	-0.1306	-0.4948	1.0000	26.7458
		0.4489	0.3746	-0.3549	7.0000	1.0000	-0.1248	4.9232
		1.0000	0.0000					
3	4	85.0402	118.8680	75.7263	0.7080	-0.1062	1.0000	16.6913
		0.2407	0.3535	-0.1906	8.4054	1.0000	-0.1154	5.6575
		1.0000	0.0000					
4	4	160.6599	73.3721	154.2849	-0.7107	-0.1462	1.0000	12.0000
		0.6826	0.9330	-0.1434	10.6712	1.0000	-0.0890	4.6486
		1.0000	0.0000					
2	3	219.7016	0.0000	0.0000	-0.6643	0.0000	1.0000	6.0000
		0.9854	5.1146	1.0000	0.0000	1.0000	-0.0532	5.1189
		0.0000	0.0000					
2	4	208.0443	0.0000	0.0000	-0.3923	0.0000	1.0000	6.0000
		0.3221	10.5505	1.0000	0.0000	1.0000	-0.0690	6.2949
		0.0000	0.0000					
1	5	128.7959	56.4134	39.0716	0.0688	-0.4463	1.0000	31.1766
		0.4530	0.1955	-0.3587	6.2148	1.0000	-0.0770	6.6386
		1.0000	0.0000					
2	5	128.6090	0.0000	0.0000	-0.5555	0.0000	1.0000	6.0000
		0.4721	10.8735	1.0000	0.0000	1.0000	-0.0242	9.1937
		1.0000	0.0000					
3	5	0.0000	0.0000	0.0000	0.5563	-0.4038	1.0000	49.5611
		0.6000	0.4259	-0.4577	12.7569	1.0000	-0.1100	7.1145
		1.0000	0.0000					
4	5	0.0000	0.0000	0.0000	0.4438	-0.2034	1.0000	40.3399
		0.6000	0.3296	-0.3153	9.1227	1.0000	-0.1805	5.6864
		1.0000	0.0000					
5	5	96.1871	93.7006	68.6860	0.0955	-0.4781	1.0000	17.8574
		0.6000	0.2723	-0.2373	9.7875	1.0000	-0.0950	6.4757
		1.0000	0.0000					
6	6	109.1904	70.8314	30.0000	0.2765	-0.3000	1.0000	16.0000
		0.1583	0.2804	-0.1994	8.1117	1.0000	-0.0675	8.2993
		0.0000	0.0000					
2	6	137.1002	0.0000	0.0000	-0.1902	0.0000	1.0000	6.0000

```
           0.4256    17.7186     1.0000     0.0000     1.0000    -0.0377     6.4281
           0.0000     0.0000
  3    6 191.1743    52.0733    43.3991    -0.2584    -0.3000     1.0000    36.0000
           0.8764     1.0248    -0.3658     4.2151     1.0000    -0.5004     4.2605
           1.0000     0.0000
 10      ! Nr of off-diagonal terms; Ediss;Ro;gamma;rsigma;rpi;rpi2
  1  2   0.0464   1.8296   9.9214   1.0029  -1.0000  -1.0000   0.0000
  2  3   0.0403   1.6913  10.4801   0.8774  -1.0000  -1.0000   0.0000
  2  4   0.0524   1.7325  10.1306   0.9982  -1.0000  -1.0000 295.0000
  1  3   0.1028   1.9277   9.1521   1.3399   1.1104   1.1609 632.0000
  1  4   0.2070   1.7366   9.5916   1.2960   1.2008   1.1262 650.0000
  3  4   0.0491   1.7025  10.6101   1.3036   1.1276   1.0173 880.0000
  2  6   0.0470   1.6738  11.6877   1.1931  -1.0000  -1.0000   0.0000
  3  6   0.1263   1.8163  10.6833   1.6266   1.2052  -1.0000   0.0000
  1  5   0.1408   1.8161   9.9393   1.7986   1.3021   1.4031   0.0000
  2  5   0.0895   1.6239  10.0104   1.4640  -1.0000  -1.0000   0.0000
 62      ! Nr of angles;at1;at2;at3;Thetao,o;ka;kb;pv1;pv2
  1  1  1  74.0317  32.2712   0.9501   0.0000   0.1780  10.5736   1.0400
  1  1  2  70.6558  14.3658   5.3224   0.0000   0.0058   0.0000   1.0400
  2  1  2  76.7339  14.4217   3.3631   0.0000   0.0127   0.0000   1.0400
  1  2  2   0.0000   0.0000   6.0000   0.0000   0.0000   0.0000   1.0400
  1  2  1   0.0000   3.4110   7.7350   0.0000   0.0000   0.0000   1.0400
  2  2  2   0.0000  27.9213   5.8635   0.0000   0.0000   0.0000   1.0400
  1  1  3  65.1700   8.0170   7.5000   0.0000   0.2028  10.0000   1.0400
  3  1  3  71.7582  26.7070   6.0466   0.0000   0.2000   0.0000   1.8525
  1  1  4  65.4228  43.9870   1.5602   0.0000   0.2000  10.0000   1.8525
  3  1  4  73.7046  23.8131   3.9811   0.0000   0.2000   0.0000   1.8525
  4  1  4  65.6602  40.5852   1.8122   0.0000   0.2000   0.0000   1.8525
  2  1  3  56.4426  17.6020   5.3044   0.0000   0.9699   0.0000   1.1272
  2  1  4  71.0777   9.1462   3.4142   0.0000   0.9110   0.0000   1.0400
  1  2  4   0.0000   0.0019   6.3000   0.0000   0.0000   0.0000   1.0400
  1  3  1  72.1018  38.4720   1.3926   0.0000   0.4785   0.0000   1.2984
  1  3  3  89.9987  44.9806   0.5818   0.0000   0.7472   0.0000   1.2639
  1  3  4  70.3281  12.9371   7.5000   0.0000   0.7472   0.0000   1.2639
  3  3  3  84.2807  24.1938   2.1695 -10.0000  0.7472   0.0000   1.2639
  3  3  4  84.2585  44.1039   0.9185   0.0000   0.7472   0.0000   1.2639
  4  3  4  74.2312  25.7005   4.3943   0.0000   0.7472   0.0000   1.2639
  1  3  2  89.0416  36.9460   0.4569   0.0000   2.7636   0.0000   2.0494
  2  3  3  81.1709   4.2886   6.5904   0.0000   3.0000   0.0000   1.2618
  2  3  4  75.9203  44.9675   0.8889   0.0000   3.0000   0.0000   1.2618
  2  3  2  82.2020  12.7165   3.9296   0.0000   0.2765   0.0000   1.0470
  1  4  1  68.3788  18.3716   1.8893   0.0000   2.4132   0.0000   1.3993
  1  4  3  86.5585  37.6814   1.1611   0.0000   1.7325   0.0000   1.0440
  1  4  4  74.4818  12.0954   7.5000   0.0000   1.7325   0.0000   1.0440
```

3	4	3	78.5850	44.3389	1.3239	-26.2246	1.7325	40.0000	1.0440
3	4	4	77.6245	32.0866	1.8889	-0.9193	1.7325	0.0000	1.0440
4	4	4	66.4718	15.9087	7.5000	0.0000	1.7325	0.0000	1.0440
1	4	2	90.0000	33.6636	1.1051	0.0000	0.2638	0.0000	1.1376
2	4	3	83.8493	44.9000	1.3580	0.0000	0.5355	0.0000	2.5279
2	4	4	78.7452	24.2010	3.7481	0.0000	0.5355	0.0000	2.5279
2	4	2	55.8679	14.2331	2.9225	0.0000	0.2000	0.0000	2.9932
1	2	3	0.0000	0.0019	6.0000	0.0000	0.0000	0.0000	1.0400
1	2	4	0.0000	0.0019	6.0000	0.0000	0.0000	0.0000	1.0400
1	2	5	0.0000	0.0019	6.0000	0.0000	0.0000	0.0000	1.0400
3	2	3	0.0000	0.0019	6.0000	0.0000	0.0000	0.0000	1.0400
3	2	4	0.0000	0.0019	6.0000	0.0000	0.0000	0.0000	1.0400
4	2	4	0.0000	0.0019	6.0000	0.0000	0.0000	0.0000	1.0400
2	2	3	0.0000	0.0019	6.0000	0.0000	0.0000	0.0000	1.0400
2	2	4	0.0000	0.0019	6.0000	0.0000	0.0000	0.0000	1.0400
1	1	5	74.4180	33.4273	1.7018	0.1463	0.5000	0.0000	1.6178
1	5	1	79.7037	28.2036	1.7073	0.1463	0.5000	0.0000	1.6453
2	1	5	63.3289	29.4225	2.1326	0.0000	0.5000	0.0000	3.0000
1	5	2	85.9449	38.3109	1.2492	0.0000	0.5000	0.0000	1.1000
1	5	5	85.6645	40.0000	2.9274	0.1463	0.5000	0.0000	1.3830
2	5	2	83.8555	5.1317	0.4377	0.0000	0.5000	0.0000	3.0000
2	5	5	97.0064	32.1121	2.0242	0.0000	0.5000	0.0000	2.8568
6	6	6	69.3456	21.7361	1.4283	0.0000	-0.2101	0.0000	1.3241
2	6	6	75.6168	21.5317	1.0435	0.0000	2.5179	0.0000	1.0400
2	6	2	78.3939	20.9772	0.8630	0.0000	2.8421	0.0000	1.0400
3	6	6	70.3016	15.4081	1.3267	0.0000	2.1459	0.0000	1.0400
2	6	3	73.8232	16.6592	3.7425	0.0000	0.8613	0.0000	1.0400
3	6	3	90.0344	7.7656	1.7264	0.0000	0.7689	0.0000	1.0400
6	3	6	22.1715	3.6615	0.3160	0.0000	4.1125	0.0000	1.0400
2	3	6	83.7634	5.6693	2.7780	0.0000	1.6982	0.0000	1.0400
3	3	6	73.4663	25.0761	0.9143	0.0000	2.2466	0.0000	1.0400
2	2	6	0.0000	47.1300	6.0000	0.0000	1.6371	0.0000	1.0400
6	2	6	0.0000	31.5209	6.0000	0.0000	1.6371	0.0000	1.0400
3	2	6	0.0000	31.0427	4.5625	0.0000	1.6371	0.0000	1.0400
2	2	5	0.0000	0.0019	6.0000	0.0000	0.0000	0.0000	1.0400

31 ! Nr of torsions;at1;at2;at3;at4;;V1;V2;V3;V2(BO);vconj;n.u;n

1	1	1	1	0.0000	48.4194	0.3163	-8.6506	-1.7255	0.0000	0.0000
1	1	1	2	0.0000	63.3484	0.2210	-8.8401	-1.8081	0.0000	0.0000
2	1	1	2	0.0000	45.2741	0.4171	-6.9800	-1.2359	0.0000	0.0000
0	1	2	0	0.0000	0.0000	0.0000	0.0000	0.0000	0.0000	0.0000
0	2	2	0	0.0000	0.0000	0.0000	0.0000	0.0000	0.0000	0.0000
0	1	3	0	1.7254	86.0769	0.3440	-4.2330	-2.0000	0.0000	0.0000
0	2	3	0	0.0000	0.1000	0.0200	-2.5415	0.0000	0.0000	0.0000
0	3	3	0	1.2314	116.5137	0.5599	-4.1412	0.0000	0.0000	0.0000

0	1	4	0	-1.3258	149.8644	0.4790	-7.1541	-2.0000	0.0000	0.0000
0	2	4	0	0.0000	0.1000	0.0200	-2.5415	0.0000	0.0000	0.0000
0	3	4	0	1.3168	57.0732	0.2679	-4.1516	-2.0000	0.0000	0.0000
0	4	4	0	2.0000	75.3685	-0.7852	-9.0000	-2.0000	0.0000	0.0000
0	1	1	0	0.0930	18.6070	-1.3191	-9.0000	-1.0000	0.0000	0.0000
4	1	4	4	-2.0000	20.6655	-1.5000	-9.0000	-2.0000	0.0000	0.0000
0	1	5	0	4.0885	78.7058	0.1174	-2.1639	0.0000	0.0000	0.0000
0	5	5	0	-0.0170	-56.0786	0.6132	-2.2092	0.0000	0.0000	0.0000
0	2	5	0	0.0000	0.0000	0.0000	0.0000	0.0000	0.0000	0.0000
0	6	6	0	0.0000	0.0000	0.1200	-2.4426	0.0000	0.0000	0.0000
0	2	6	0	0.0000	0.0000	0.1200	-2.4847	0.0000	0.0000	0.0000
0	3	6	0	0.0000	0.0000	0.1200	-2.4703	0.0000	0.0000	0.0000
1	1	3	3	1.2707	21.6200	1.5000	-9.0000	-2.0000	0.0000	0.0000
1	3	3	1	-1.8804	79.9255	-1.5000	-4.1940	-2.0000	0.0000	0.0000
3	1	3	3	-2.0000	22.5092	1.5000	-8.9500	-2.0000	0.0000	0.0000
1	4	4	3	0.1040	70.1152	0.5284	-3.5026	-2.0000	0.0000	0.0000
1	1	3	4	1.2181	119.6186	-1.5000	-7.0635	-2.0000	0.0000	0.0000
2	1	3	4	-2.0000	156.6604	1.1004	-7.3729	-2.0000	0.0000	0.0000
1	3	4	3	2.0000	96.6281	-1.5000	-3.8076	-2.0000	0.0000	0.0000
1	1	4	2	-2.0000	147.2445	-1.5000	-7.0142	-2.0000	0.0000	0.0000
1	1	4	3	-2.0000	47.8326	-1.5000	-9.0000	-2.0000	0.0000	0.0000
2	3	4	3	-0.2997	152.9040	-1.5000	-4.4564	-2.0000	0.0000	0.0000
2	4	4	3	0.1040	70.1152	0.5284	-3.5026	-2.0000	0.0000	0.0000

9 ! Nr of hydrogen bonds;at1;at2;at3;Rhb;Dehb;vhb1

3	2	3	2.1845	-2.3549	3.0582	19.1627
3	2	4	1.6658	-3.8907	3.0582	19.1627
4	2	3	1.8738	-3.5421	3.0582	19.1627
4	2	4	1.8075	-4.1846	3.0582	19.1627
3	2	5	2.6644	-3.0000	3.0000	3.0000
4	2	5	4.0476	-3.0000	3.0000	3.0000
5	2	3	2.1126	-4.5790	3.0000	3.0000
5	2	4	2.2066	-5.7038	3.0000	3.0000
5	2	5	1.9461	-4.0000	3.0000	3.0000
